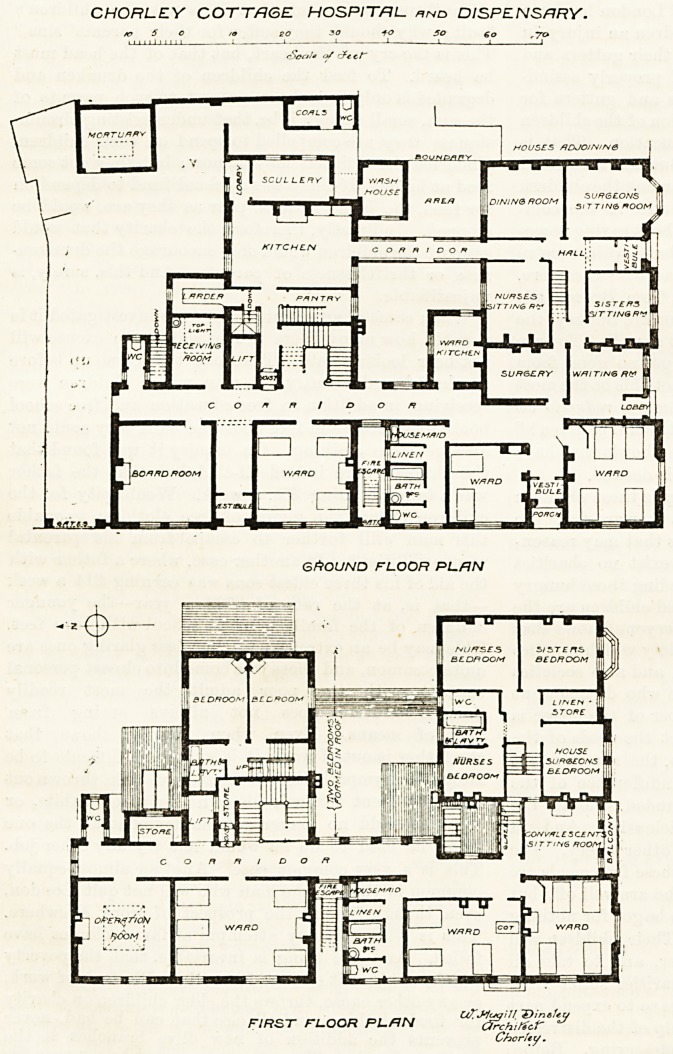# Hospital Construction

**Published:** 1900-01-06

**Authors:** 


### The Institutional Workshop.

### HOSPITAL CONSTRUCTION.

### CHORLEY COTTAGE HOSPITAL.

In criticising this hospital it is essential to remember that the building as it now stands represents three different dates, and was intended to serve two purposes

—a dispensary and a hospital—and as the site was a limited one with no possibility of extension except northwards and a few yards westward, the best had to be made of this cramped space or the hospital removed elsewhere. Probably the funds would not allow the latter alternative, and it may be admitted that the former has been fairly well carried out. True, the wards are innocent of proper direct cross ventilation. Indeed the only room in the hospital which has windows on diametrically opposite sides is the kitchen; and there are two wards in which windows are placed on two sides of the room but not opposite to each other. The original building was a dispensary, and was built in 1891. Afterwards, in 1893, wards, offices and a mortuary were added, and finally the kitchen department was remodelled, more wards were put up, with a new board-room, operating-room, lifts, cellars, &c. The entrance-hall is in the east side, and to the left on entering is the board-room. The receiving-room is almost opposite, and the lift and staircase adjoin the receiving-room. On the right is a ward for four beds; next to this a fire escape-room, and then the sanitary arrangements. The housemaid's closet, the linen-room, and bath-room have only borrowed light; but the closet is sufficiently lighted. No part is cut off from the building by a ventilating passage, so that one of the most important points in hospital construction has either been overlooked, or it has been found impossible to carry out owing to the site. This drawback and the absence of proper cross-ventilation in the wards makes us ask whether it would not have been possible to remove the hospital to a new site permitting of thoroughly satisfactory arrangements. Passing south of the bath-room we come to a ward for two beds, then an additional entrance to the hospital and another ward for four beds. As there is another entrance facing south we may ask again why the porch and vestibule were not swept away and one large ward formed? Had this been done more light and better ventilation would have been obtained and also space for another bed. The rest of the ground-floor is taken up with the kitchen department, surgery, waiting-room, and sitting-rooms for the staff. The first floor is almost the same as the ground floor, save that the operating-room takes the place of the board-room, and that bed-rooms for the staff are over the kitchen and the sitting-rooms. The accommodation is for 23 patients. Mr. W. H. Dinsley is the architect of the new work.

## Figures and Tables

**Figure f1:**